# Persistence and emergence of new neuropsychological deficits following SARS-CoV-2 infection: A follow-up assessment of the Geneva COVID-COG cohort

**DOI:** 10.7189/jogh.14.05008

**Published:** 2024-03-08

**Authors:** Philippe Voruz, Isabele Jacot de Alcântara, Anthony Nuber-Champier, Alexandre Cionca, Delphine Guérin, Gilles Allali, Lamyae Benzakour, Patrice H Lalive, Karl-Olof Lövblad, Olivia Braillard, Umberto Nencha, Mayssam Nehme, Matteo Coen, Jacques Serratrice, Jean-Luc Reny, Jérôme Pugin, Idris Guessous, Basile N Landis, Frédéric Assal, Julie A Péron

**Affiliations:** 1 Clinical and Experimental Neuropsychology Laboratory, Faculty of Psychology, University of Geneva, Geneva, Switzerland; 2 Department of Clinical Neurosciences, Neurology Department, Geneva University Hospitals, Switzerland; 3 Faculty of Medicine, University of Geneva, Switzerland; 4 Leenaards Memory Center, Lausanne University Hospital and University of Lausanne, Lausanne, Switzerland; 5 Psychiatry Department, Geneva University Hospitals, Switzerland; 6 Diagnostic and Interventional Neuroradiology Department, Geneva University Hospitals, Switzerland; 7 Division and Department of Primary Care, Geneva University Hospitals, Switzerland; 8 Geneva Memory Center, Division of Geriatrics, Geneva University Hospitals, Switzerland; 9 Division of General Internal Medicine, Department of Medicine, Geneva University Hospitals and Geneva University, Switzerland; 10 Intensive Care Department, Geneva University Hospitals, Switzerland; 11 Rhinology-Olfactology Unit, Otorhinolaryngology Department, Geneva University Hospitals, Switzerland

## Abstract

**Background:**

Despite numerous observations of neuropsychological deficits immediately following severe acute respiratory syndrome coronavirus 2 (SARS-CoV-2) infection, little is known about what happens to these deficits over time and whether they are affected by changes in fatigue and any psychiatric symptoms. We aimed to assess the prevalence of neuropsychological deficits at 6–9 months and again at 12–15 months after coronavirus disease 2019 (COVID-19) and to explore whether it was associated with changes in fatigue and psychiatric symptoms.

**Methods:**

We administered a series of neuropsychological tests and psychiatric questionnaires to 95 patients (mean age = 57.12 years, standard deviation (SD) = 10.68; 35.79% women) 222 (time point 1 (T1)) and 441 (time point 2 (T2)) days on average after infection. Patients were categorised according to the severity of their respiratory COVID-19 symptoms in the acute phase: mild (no hospitalisation), moderate (conventional hospitalisation), and severe (hospitalisation in intensive care unit (ICU) plus mechanical ventilation). We ran Monte-Carlo simulation methods at each time point to generate a simulated population and then compared the cumulative percentages of cognitive disorders displayed by the three patient subgroups with the estimated normative data. We calculated generalised estimating equations for the whole sample to assess the longitudinal associations between cumulative neuropsychological deficits, fatigue, and psychiatric data (anxiety, depressive symptoms, posttraumatic stress disorder, and apathy).

**Results:**

Most participants (>50%) exhibited a decrease in their neuropsychological impairments, while approximately 25% showed an escalation in these cognitive deficits. At T2, patients in the mild subgroup remained free of accumulated neuropsychological impairments. Patients with moderate severity of symptoms displayed a decrease in the magnitude of cumulative deficits in perceptual and attentional functions, a persistence of executive, memory and logical reasoning deficits, and the emergence of language deficits. In patients with severe symptoms, perceptual deficits emerged and executive deficits increased, while attentional and memory deficits remained unchanged. Changes in executive functions were significantly associated with changes in depressive symptoms, but the generalised estimating equations failed to reveal any other significant effect.

**Conclusion:**

While most cumulative neuropsychological deficits observed at T1 persisted and even worsened over time in the subgroups of patients with moderate and severe symptoms, a significant proportion of patients, mainly in the mild subgroup, exhibited improved performances. However, we identified heterogeneous neuropsychological profiles both cross-sectionally and over time, suggesting that there may be distinct patient phenotypes. Predictors of these detrimental dynamics have yet to be identified.

Recent studies have highlighted the persistence of neuropsychological deficits up to 12 months after infection with severe acute respiratory syndrome coronavirus 2 (SARS-CoV-2), regardless of the severity of respiratory symptoms in the acute phase [1–3]. These deficits mainly concern memory, attention, and executive functions, suggesting that coronavirus disease 2019 (COVID-19) has long-term consequences beyond the effects of hospitalisation in an intensive care unit (ICU). However, these studies assessed the results of individual neuropsychological tests without considering collinearity (i.e. correlations between neuropsychological tests), cumulative neuropsychological deficits [4,5], or the inherent limitations of cumulative neuropsychological testing in an assessment [4]. The use of multiple neuropsychological tests assessing multiple cognitive functions (e.g. memory, executive, or attentional) can lead to biases such as the identification of isolated neuropsychological deficits in up to 9% of patients considered to be healthy [4]. To the best of our knowledge, only one previous study, carried out by our group, has so far taken this statistical issue into account. Results from the Geneva COVID-COG cohort at 6–9 months post-infection revealed a significantly higher accumulation of neuropsychological deficits for memory, executive functions, attention, and logical reasoning, compared with a normative simulated population, in patients who had had moderate or severe symptoms in the acute phase, but not in patients who had had mild symptoms [6]. Longitudinal follow-up of very long-term cognitive disorders following SARS-CoV-2 infection is still lacking, as are studies seeking to identify their potential predictors.

There is currently no consensus in the literature on the potential effects of secondary variables (e.g. psychiatric symptoms or fatigue) on the prevalence of neuropsychological deficits following SARS-CoV-2 infection. Some authors have highlighted associations between psychiatric variables (e.g. apathy, anxiety, or depressive symptoms) and performances on neuropsychological tests [7,8], while others have not [9]. Some sociodemographic and clinical characteristics (e.g. age, severity of infection, or medical comorbidities), as well as the potential existence of distinct patient phenotypes that go beyond the severity of the acute infection, may explain these divergent results [9,10]. Recent data point to two phenotypes of patients with dissociated neuropsychological symptoms: symptoms similar to those of chronic fatigue and symptoms associated with the development of a neurodegenerative cascade. Concerning the first phenotype, the literature on pathologies such as myalgic encephalomyelitis/chronic fatigue syndrome (ME/CFS) [11,12] suggests an interesting parallel, as neuropsychological deficits have been associated with chronic fatigue symptoms. In particular, a meta-analysis revealed neuropsychological deficits in patients with ME/CFS, predominantly in the form of executive and attentional deficits that tended to improve over time [11]. These types of deficits have also been observed following SARS-CoV-2 infection, but there is currently no data on how they change over time. Concerning the second phenotype, viral infectious diseases have recently been shown to be major risk factors for the development of neurodegenerative diseases (e.g. influenza for Alzheimer disease or Parkinson disease [13]). Regarding SARS-CoV-2 infection, examination of the neurocognitive trajectories of a subgroup of patients beyond the acute phase has led authors to formulate hypotheses about the impact of this infection on neurocognitive aging [14,15]. Given the persistence of cognitive impairment – predominantly episodic memory disorders, instrumental disorders (language, perception, praxis) and anosognosia [6,10], together with neuroinflammatory markers [16–18] and neurostructural changes [19,20] – hypotheses of accelerated brain aging are being intensively investigated, but rarely in cohorts of patients with no comorbidities before the infection that might also influence these processes [14,15].

In this context, our main aim was to perform a follow-up assessment of the Geneva COVID-COG cohort [6] to longitudinally assess the prevalence of cumulative neuropsychological deficits 12–15 months after SARS-CoV-2 infection, compared with the prevalence of cumulative deficits at 6–9 months. Our second aim was to assess whether changes in the cumulative neuropsychological deficits over time were associated with changes in self-reported psychiatric symptoms and fatigue [8,10,21]. We expected to observe changes in neuropsychological performances that were relatively independent of the severity of the respiratory form in the acute phase. Although we continued to categorise patients according to acute-phase severity, in accordance with the original COVID-COG protocol designed in April 2020, we have learned that the severity of the respiratory form in the acute phase may be a contributing risk factor, but was not the only determining one, based on the literature that has emerged since the start of the project [10]. We therefore also explored the directions of the long-term dynamics and their associated factors. The neurodegenerative trajectory hypothesis [14,15] predicted a long-term deterioration in neuropsychological functions (especially memory, instrumental, and executive functions) in some patients, the persistence or emergence of anosognosia at 12–15 months post-infection, as well as a significant relationships between apathy and both episodic memory and instrumental disorders [9]. The chronic fatigue trajectory hypothesis [11,12] predicted stability or even a slight improvement in neuropsychological performance over the long term, with effects mainly on executive and attentional functions at 12–15 months post-infection in some patients. Executive and attentional deficits would thus be significantly correlated with psychiatric symptoms, especially anxiety and depressive symptoms, as well as with self-reported fatigue [11].

## METHODS

### Participants

We initially considered 4000 patients of Geneva University Hospitals (HUG) who had been infected with SARS-CoV-2 between March 2020 and May 2021 for potential inclusion. To be part of the research, their infection had to have been confirmed by positive reverse transcriptase polymerase chain reaction (RT/PCR) results from a nasopharyngeal swab and/or by positive serological results. We applied the following exclusion criteria at time point 1 (T1): history of neurological issues, psychiatric disorders (two of the included participants had had an episode of depression >10 years before their SARS-CoV-2 infection), oncological and neurodevelopmental pathologies, pregnancy, and age >80 years. Participants were recruited either via admission lists provided by the CoviCare program [22] or via another study performed at HUG [23].

### General procedure and ethics

We fully described the study to the participants, who then provided their written informed consent. The study was conducted in accordance with the Declaration of Helsinki, and the cantonal ethics committee of Geneva (CCER-02186) approved the study protocol.

### Neurological and neuropsychological assessments

Three board-certified clinical neurologists (FA, GA, and UN) conducted a neurological assessment. Three certified psychologists (PV, ANC, IJA) under the supervision of a board-certified clinical neuropsychologist (JP) administered a series of neuropsychological tests and questionnaires to patients, who were all fluent in written and spoken French (**Table 1**). We had normative data validated in a French-speaking population for all these assessment tools. On average, the neuropsychological assessment lasted 180 minutes at both T1 and time point 2 (T2) (6–9 months and 12–15 months post-infection, respectively). Patients also responded to online questionnaires using Qualtrics software (Qualtrics, Provo, UT, USA); mean time taken was 60 minutes.

**Table 1:** Domains and functions measured by the neuropsychological tests used in the COVID-COG protocol at T1 (6–9 months post-infection) and T2 (follow-up at 12–15 months post-infection)

Domain	Functions	Names of tests
Perception	Object perception	Incomplete Letters and Object Decision tests from Visual Object and Space Perception battery [24]
	Spatial perception	Number Location and Cube Analysis tests from Visual Object and Space Perception battery [24]
Ideomotor praxis		Moroni praxis battery [25]
Language	Semantic processing: naming and repetition	Semantic image matching, semantic word matching, oral picture naming, word repetition, and nonword repetition from BECLA battery [26]
Executive functions	Inhibition	Stroop task from GREFEX battery [27]
	Mental flexibility	Trail Making Test from GREFEX battery [27]
	Verbal fluency	Categorical and Verbal Fluency from GREFEX battery [27]
	Verbal working memory	Digit Span Backward from WMS-III [28]
	Visuospatial working memory	Backward Corsi test from WAIS-IV [29] and Test of Attentional Performance [30]
Attention	Phasic alertness; divided and sustained attention; incompatibility	Test of Attentional Performance [30]
Memory	Episodic verbal	Grober and Buschke free/cued recall paradigm [31]
	Episodic visuospatial	Delayed recall of Rey-Osterrieth Complex Figure [32]
	Anosognosia for memory dysfunction	Self-appraisal discrepancy score for each memory test [10]
Logical reasoning		Matrix Reasoning and Visual Puzzles subtests from WAIS-IV [28]

BECLA – Batterie d'Evaluation Cognitive du Langage (Cognitive Language Evaluation Battery), WMS-III – Wechsler Memory Scale – III, WAIS-IV – Wechsler Adult Intelligence Scale

### Psychiatric symptoms and fatigue

We measured the following psychiatric variables at each time point: depression with the Beck Depression Inventory-Second Edition (BDI) [33], anxiety with the State-Trait Anxiety Inventory (STAI) [34], apathy and its distinct subtypes with the Apathy Motivation Index (AMI) [35], and posttraumatic stress disorder (PTSD) with the Posttraumatic Stress Disorder Checklist for the Diagnostic and Statistical Manual (PSDC for DSM-5) [36]. Fatigue was measured with the French version of the Fatigue Impact Scale (EMIF-SEP) [37].

### Measure of symptom validity

At each time point, we used the Behavior Rating Inventory of Executive Function for Adults (BRIEF-A) [38] and Wechsler Adult Intelligence Scale–Fourth Edition (WAIS-IV) [38] digit spans to assess the validity of the patients’ symptoms (i.e. congruence), and the presence of any symptoms that needed to be considered with care [6].

### Statistical analyses

To explore the frequency of abnormally low neuropsychological scores at 6–9 months and 12–15 months post-infection (aim 1), we adopted the following procedure. For each validated neuropsychological test, patients’ performances were first compared with normative data for that tool collected from a reference sample. More specifically, raw scores were converted to standardised scores (t- and z-scores, percentiles, or standard scores), which were adjusted for age, education, and gender using published normative data. We used a conservative threshold (<5^th^ percentile) as defined by the Swiss Society of Neuropsychology to establish the difference between patients’ deficits and normative data. To find out whether the frequency of the deficits observed in our patient cohort was higher than that expected in a normative population, and to compensate for the number of tests (Type 1 error), we then used the Monte Carlo simulation program (i.e. estimated base rates), with a framework developed by Crawford et al. [5] and previously validated with the Geneva COVID-COG cohort [6]. The estimation of the baseline rates of low scores, based on test intercorrelations for the whole COVID-19 sample at T1, allowed us to estimate the percentage of the normative population who would exhibit one or more, two or more, three or more, or four or more abnormally low scores, applying a conservative threshold (<5^th^ percentile) [5]. We then carried out six stages of analysis. First, we pooled the scores according to seven cognitive functions, based on theoretical models and/or test batteries: episodic memory model [39] and Memory NEo-Structural Inter-Systemic model (MNESIS) model [40] for memory; latent model of executive functions [41] and GREFEX battery [27] for executive functions; test of Attentional Performance battery [30] for attentional abilities; subtests of the Visual Object and Space Perception battery [24] for perceptual abilities; subtests of the WAIS-IV [28] for logical reasoning; subtests of the Cognitive Language Evaluation Battery (Batterie d'Evaluation Cognitive du Langage) (BECLA) [26] for language; and subtests of a praxis battery [25] for ideomotor praxis (detailed scores provided in Table S2 in the **Online Supplementary Document**). Second, we calculated correlation matrices of raw scores for each function. Third, we entered the results of the correlation matrices into a generic program: PercentAbnormKtests [5] with a conservative threshold (<5^th^ percentile). It should be noted that the results for the 95 patients were generally comparable to the results of a previous simulation carried out on 121 patients [6]. Fourth, we summed scores below the conservative threshold for each patient and each function. Fifth, we calculated cumulative percentages of patients in the total sample and each subgroup (mild, moderate, and severe) with at least one lower test score. Sixth and last, we compared these cumulative percentages first with the estimated scores of the normative population obtained at Stage 3 using binomial distribution probability analyses, as suggested by Crawford et al. [5], then with the estimated baseline rate used to specify the sample *P*-value: baseline estimate vs mild group, baseline estimate vs moderate group, and baseline estimate vs severe group. Finally, we used a Benjamini-Hochberg false discovery rate (FDR) correction [42] with a *P*-value set at .05 for each group comparison on each of the functions we assessed.

To assess the longitudinal relationship between the prevalence of cumulative neuropsychological deficits and secondary variables (psychiatric symptoms and fatigue) (aim 2), we applied the following procedure. We calculated independent generalised estimating equations (GEEs) in SPSS, version 28.0 (IBM, Armonk, New York, USA) to assess the relationships between neuropsychological data and our secondary variables of interest (depressive symptoms, anxiety, apathy, PTSD, and fatigue) over time. Sociodemographic data (age, gender, and education level) and the severity of the infection in the acute phase were added to the models. We calculated a separate model for each of the seven dependent cognitive domains (memory, executive, perceptual, language, praxis, logical reasoning, attention). Given the ordinal distribution of our dependent variables and the presence of repeated nonparametric continuous measures as predictors, we ran GEE ordinal probit models solely with the main effects of the predictors.

## RESULTS

### Participants

**Figure 1:**
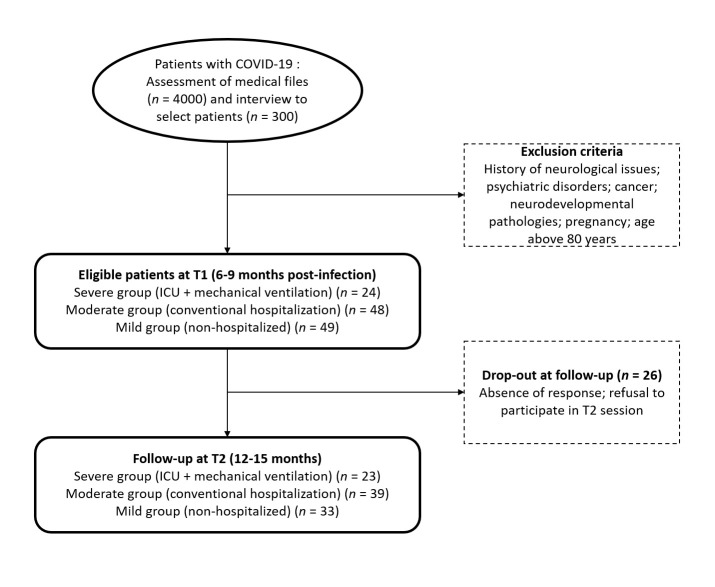
Flowchart of the study. ICU – intensive care unit.

After screening 4000 medical records at HUG according to our exclusion criteria, we found 300 patients to be eligible, of which 121 agreed to participate in the study at a mean 222.46 (standard deviation (SD) = 42.93) days after infection (T1), while 95 of these also agreed to participate in the follow-up at a mean 441.34 (SD = 54.3) days after infection (T2) (**Figure 1**). To gain an unbiased assessment of the prevalence of deficits, we only analyzed data on the 95 patients who attended the sessions at both time points. The reasons for the nonparticipation of the 26 individuals (21.48% of the sample) in the follow-up were lack of response, and refusal to take part (reasons given: lack of time, participants did not see the need for a follow-up assessment).

We divided patients into three subgroups according to the severity of their infection in the acute phase (**Table 2**): 33 had tested positive but had not been hospitalised (mild symptoms group); 39 had been hospitalised but did not require mechanical ventilation (moderate symptoms group) (9.86 (SD = 8.09) days of hospitalisation), and 23 had been admitted to ICU during the acute phase of the infection and undergone mechanical ventilation (severe symptoms group) (37.25 (SD = 25.71) days of hospitalisation). The mild and moderate subgroups were matched (and verified through nonparametric analysis) with patients in the severe subgroup on the following criteria: median age (mild = 55 years; moderate = 56 years; severe = 60 years), education level, language (all were French-speaking Swiss citizens or residents of the French part of Switzerland), clinical variables (except for smoker status, which was significantly higher in the mild subgroup), and number of days since infection (T1: effect size (*H* )= 0.95; *P* = 0.623, T2: *H* = 0.80; *P* = 0.671). Moreover, based on information provided by participants, we retrospectively ascertained which patients had performed risky occupations (as defined by the Swiss Confederation) during the pandemic (e.g. health care professional or supermarket cashier). Analysis did not reveal any differences in the percentage of at-risk occupations across the groups (sample = 87.88%, *H* = 0.524; *P* = 0.334). The frequency of each of the different clinical symptoms experienced by patients during the acute phase is available in Table S1 in the **Online Supplementary Document**.

**Table 2:** Sociodemographic data and relevant medical history*

	Total sample (n = 95)	Mild subgroup (n = 33)	Moderate subgroup (n = 39)	Severe subgroup (n = 23)	*P*-value†
Age in years, mean (SD) (range)	57.12 (10.68), (36-38)	54.24 (8.31), (37–69)	56.46 (10.65), (36–75)	62.35 (12.23), (38–78)	NS
Education level, (1-3), mean (SD)†	2.63 (0.55)	2.73 (0.45)	2.62 (0.59)	2.52 (0.59)	NS
Gender – women, %	35.79	42.42	38.46	21.74	NS
Handedness – right-handed, %	95.79	96.97	94.87	95.65	NS
Days of hospitalisation, mean (SD)	19.82 (20.70)	-	9.86 (8.09)	37.25 (25.71)	NA
Diabetes, %	7.37	0	5.13	21.74	NS
Smoking, %	5.26	15.15	0	0	0.007†
History of respiratory disorders, %	5.26	15.15	0	0	NS
History of cardiovascular disorders, %	17.89	15.15	15.38	26.09	NS
History of neurological disorders, %	0	0	0	0	NS
History of psychiatric disorders, %	4.21	6.06	0	0	NS
History of cancer, %	0	0	0	0	NS
History of severe immunosuppression, %	0	0	0	0	NS
History of developmental disorders, %	0	0	0	0	NS
Chronic renal failure, %	0	0	0	0	NS
Sleep apnea syndrome, %	15.79	9.09	15.38	26.09	NS

SD – standard deviation, NA – not applicable, NS – not significant

*Mild: patients not hospitalised for COVID-19; moderate: patients hospitalised without mechanical ventilation for COVID-19; severe: patients hospitalised in intensive care unit with mechanical ventilation for COVID-19.

†Statistical analysis performed: Kruskal-Wallis or χ^2^.

‡Level 1 is equivalent to compulsory Swiss schooling (11 years of study); Level 2 is equivalent to a vocational diploma (11-12 years of study); and Level 3 is equivalent to the Matura high-school diploma and higher education (>12 years of study).

### Changes in neuropsychological deficits for whole sample

When we examined changes in neuropsychological deficits test by test, we observed an overall improvement in performance in 54.66% of the sample, while there was decline in performance in 25.63% of the sample, and neuropsychological deficits persisted in 10.48% of patients. Finally, 9.22% of the sample exhibited no neuropsychological deficits at either time point (T1 and T2) (**Table 3**). Cumulative neuropsychological deficits and their changes over time (at 6–9 months and 12–15 months post-infection) in each subgroup are shown in Table S2 in the **Online Supplementary Document**.

**Table 3:** Descriptive data for the whole sample and each subgroup (mild, moderate, and severe) for changes in neuropsychological deficits between time point 1 and time point 2, considering the neuropsychological tests separately, not cumulatively

	Whole sample (n = 95)	Mild subgroup (n = 33)	Moderate subgroup (n = 39)	Severe subgroup (n = 23)
Percentage increase in total number of neuropsychological deficits (<5^th^ percentile)	25.63	30.30	20.51	26.09
Percentage decrease in total number of neuropsychological deficits (<5^th^ percentile)	54.66	48.48	58.97	56.52
Percentage with no modification in total number of neuropsychological deficits (<5^th^ percentile)	10.48	3.03	15.38	13.04
Absence of neuropsychological deficits at both time points	9.22	18.18	5.13	4.35

### Cumulative neuropsychological deficits at 6–9 months and 12–15 months post-infection according to severity of acute-phase infection

All results below were yielded by binomial distribution probability analyses with FDR Benjamini-Hochberg correction.

#### Perceptual functions

**Figure 2:**
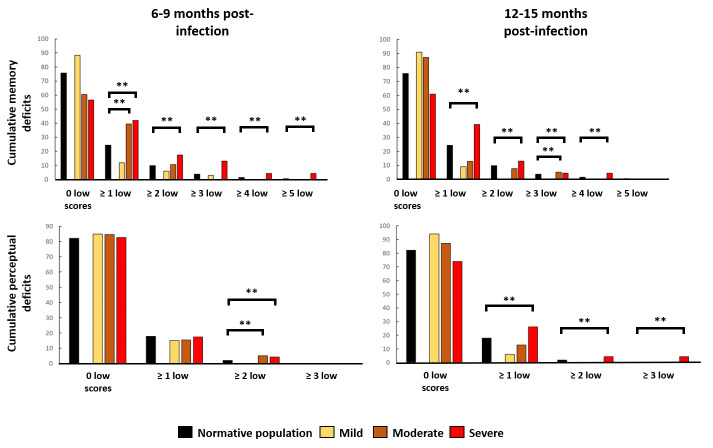
Cumulative memory and perceptual deficits at 6–9 and 12–15 months post-infection. **Panel A.** Significantly higher cumulative percentages of memory deficits at 6–9 months post-infection for the severe (one, two, three, four, and five or more cumulative deficits) and moderate (one or more cumulative deficits) subgroups, compared with a normative population. **Panel B.** Significantly higher cumulative percentages of memory deficits at 12–15 months post-infection for the severe (one, two, three, and four or more cumulative deficits) and moderate (three or more cumulative deficits) subgroups, compared with the normative population. **Panel C.** Significantly higher cumulative percentages of perceptual deficits at 6–9 months post-infection for the severe (one, two or more cumulative deficits) and moderate (two or more cumulative deficits) subgroups, compared with the normative population. **Panel D.** Significantly higher cumulative percentages of perceptual deficits at 12–15 months post-infection solely for the severe subgroup (one, two, and three or more cumulative deficits), compared with the normative population.

At 6–9 months post-infection, the moderate and severe subgroups differed significantly from the normative population on perceptual functions, with two or more abnormally low scores (moderate: +4.15%; *P* = 0.001, severe: +3.37%; *P* = 0.001), while all comparisons with the normative population on cumulative percentages were nonsignificant for the mild subgroup (*P* > 0.05). At 12–15 months post-infection, the severe subgroup differed significantly from the normative population on one or more (+8.21%; *P* < 0.001), two or more (+3.37%; *P* = 0.001), or three or more (+4.21%; *P* < 0.001) abnormally low scores, while the mild and moderate subgroups did not differ significantly from the normative population on any cumulative percentages (*P* > 0.05). Results for perceptual functions at 6–9 months pointed to a long-term cumulative performance for the mild subgroup that was comparable to that of a normative population. By contrast, a cumulative deficit lasting more than 6–9 months was observed in both the moderate and severe subgroups. We observed a normalisation of performances at 12–15 months for the moderate subgroup, but a clear worsening for the severe subgroup (**Figure 2** and Table S2 in the **Online Supplementary Document**)

#### Ideomotor praxis

The analysis revealed no significant differences at 6–9 months post-infection between the normative population and either the total sample or the mild, moderate, and severe subgroups on ideomotor praxis (*P* > 0.05). At 12–15 months post-infection, analysis similarly failed to reveal any significant differences between the normative population and either the total sample or the mild, moderate, and severe subgroups (*P* > 0.05). These results for ideomotor functions suggested the presence of cumulative deficits comparable to those of a normative population at each time point, regardless of the severity of the infection in the acute phase (Table S2 in the **Online Supplementary Document**)

#### Language

The analysis revealed no significant differences at 6–9 months post-infection between the normative population and either the total sample or the mild, moderate, and severe subgroups (*P* > 0.05). However, we found significant difference at 12–15 months post-infection between the moderate subgroup and the normative population on three or more abnormally low scores (+1.69%; *P* = 0.004), while for the mild and severe subgroups, all cumulative percentages were nonsignificant, compared with the normative population (*P* > 0.05). These results pointed to a deterioration in language abilities in the moderate subgroup at 12–15 months post-infection (Table S2 in the **Online Supplementary Document**).

#### Executive functions

We found a significant difference at 6–9 months post-infection between the moderate subgroup and the normative population on three or more abnormally low scores (+2.08%; *P* = 0.011), while all cumulative percentages were nonsignificant for the mild and severe subgroups, compared with the normative population (*P* > 0.05). We observed a significant difference at 12–15 months post-infection between the moderate subgroup and the normative population on two or more abnormally low scores (+2.78%; *P* < 0.001), but found significant differences for the severe subgroup on one or more (+2.45%; *P* < 0.001), four or more (+1.71%; *P* = 0.003), and five or more (+3.18%; *P* < 0.001) abnormally low scores. All cumulative percentages for the mild subgroup were nonsignificant compared with the normative population (*P* > 0.05). Results for executive functions pointed to a deterioration, and therefore an increase in the accumulation of deficits. However, this could only be observed in the severe subgroup, as the moderate subgroup maintained a stable level of cumulative deficits compared with the normative population, and the mild subgroup did not have significantly greater cumulative deficits (Table S2 in the **Online Supplementary Document**).

#### Attentional functions

We found a significant difference at 6–9 months post-infection between the moderate subgroup and the normative population on four or more abnormally low scores (+1.22%; *P* = 0.021). We further observed significant differences for the severe subgroup on two or more abnormally low scores (+3.18%; *P* < 0.001), while all cumulative percentages for the mild subgroup were nonsignificant, compared with the normative population after FDR correction (*P* > 0.05). At 12–15 months post-infection, significant differences were found for the severe subgroup on three or more abnormally low scores (+1.50%; *P* = 0.003), while all cumulative percentages for the mild and moderate subgroups were nonsignificant, compared with the normative population (*P* > 0.05). Results for attentional functions suggested a normalisation in the moderate subgroup, while cumulative deficits persisted in the severe subgroup, compared with the normative population. The mild subgroup did not have a significantly higher cumulative deficit at either time point (Table S2 in the **Online Supplementary Document**).

#### Memory functions

The moderate subgroup differed significantly from the normative population on one or more abnormally low scores (+15.11%; *P* < 0.001) at 6–9 months post-infection. The severe subgroup differed significantly on one or more (+19.12%; *P* < 0.001), two or more (+7.57%; *P* < 0.001), three or more (+9.31%; *P* < 0.001), four or more (+2.90%; *P* < 0.001) and five or more (+3.82%; *P* < 0.001) abnormally low scores, while all cumulative percentages for the mild subgroup were nonsignificant, compared with the normative population (*P* > 0.05). At 12–15 months post-infection, the moderate subgroup differed significantly from the normative population on three or more abnormally low scores (+1.39%; *P* = 0.011). The severe subgroup differed significantly on one or more (+14.67%; *P* < 0.001), two or more (+3.22%; *P* < 0.001), three or more (+0.82%; *P* = 0.008), and four or more abnormally low scores (+2.90%; *P* < 0.001). All cumulative percentages for the mild subgroup were nonsignificant, compared with the normative population (*P* > 0.05). These results suggested the presence and persistence of cumulative memory deficits in the moderate and severe subgroups at each time point, while there were no significant differences between the mild subgroup and the normative population for memory functions (Table S2 in the **Online Supplementary Document** and **Figure 2**)

#### Logical reasoning

We found a significant difference between the moderate subgroup and the normative population on three or more abnormally low scores (+1.69%; *P* = 0.018) at 6–9 months post-infection, while all differences on cumulative percentages between the mild and severe subgroups and the normative population were nonsignificant (*P* > 0.05). The moderate subgroup differed significantly from the normative population on three or more abnormally low scores (+1.69%; *P* = 0.018) at 12–15 months post-infection, while all differences on cumulative percentages between the mild and severe subgroups and the normative population were nonsignificant (*P* > 0.05). These results for logical reasoning pointed to a persistent cumulative performance deficit in the moderate subgroup, but no cumulative deficits in the mild and severe subgroups at either time point (Table S2 in the **Online Supplementary Document**).

#### Longitudinal relationships between cumulative neuropsychological deficits and psychiatric data and fatigue

**Figure 3:**
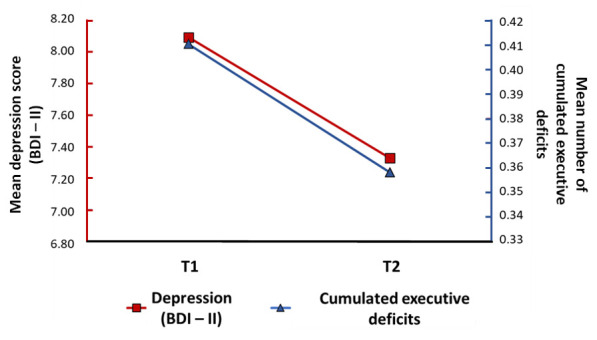
Significant longitudinal association between changes in cumulative executive function deficits and changes in self-reported depressive symptoms. BDI II – BECK depression inventory.

GEE analyses only revealed a significant relationship between changes in executive functioning and changes in depressive symptoms (Wald χ^2^ = 5.32; *P* = 0.021, CI = 0.016, 0.193), suggesting that a reduction in the prevalence of executive disorders was associated with a decrease in self-reported psychiatric symptoms. Executive functioning was also significantly associated with the severity of the acute infection (Wald χ^2^ = 7.66; *P* = 0.006), but not with age, gender, or education level. No significant longitudinal relationships were found for any of the other cognitive functions (memory, instrumental, attentional, or logical reasoning) with changes over time in either self-reported psychiatric symptoms or fatigue (*P* > 0.050) (**Figure 3**).

## DISCUSSION

More than half of the Geneva COVID-COG cohort showed a reduction in overall neuropsychological deficits. However, a significant proportion of patients (~40%) exhibited a worsening or persistence of neuropsychological deficit scores, suggesting the presence of distinct patterns of neurocognitive change following SARS-CoV-2 infection. We were therefore able to confirm the results of the previous assessment at 6–9 months, validating the use of cumulative deficits and statistical simulation methods to measure the long-term neuropsychological effects of SARS-CoV-2 infection, with two main implications.

First, our results confirmed that the neurocognitive consequences of SARS-CoV-2 infection go beyond any effect of ICU hospitalisation. More specifically, neuropsychological deficits at 6–9 and 12–15 months post-infection were found to be partially independent of the severity of the initial infection, thus supporting previous observations [1,6,9,10,43–45]. Moreover, concerning changes in the neuropsychological deficits, the results of our assessment at 12–15 months indicated a normalisation of attentional deficits in the moderate subgroup, the persistence of memory and logical reasoning symptoms in both the moderate and severe subgroups, and deteriorations in both instrumental functioning and executive functions in the moderate and severe subgroups. The latter results for the severe subgroup go against the results of studies assessing the effects of ICU that were conducted prior to the COVID-19 pandemic, as these indicated that cognitive functions improve within approximately six months of hospitalisation in ICU [46]. Additionally, the absence of cumulative neuropsychological deficits in the mild subgroup (confirming previous results at 6–9 months) [6] does not exclude the possibility of isolated neuropsychological deficits, as observed in previous studies [1,9]. These isolated deficits could have an impact on patients’ daily lives [9,47] and could be associated with brain modifications [48]. Thus, in line with our predictions, although the severity of respiratory symptoms in the acute phase may be a contributing factor for the presence of neuropsychological deficits after the infection, it is not the only predictor of what we may now call neuropsychological post-COVID-19 condition. This variable should therefore be regarded as one risk factor among others (e.g. behavioral, immunological, and/or genetic) [18].

Second, our results pointed to the probable existence of distinct clinical pathological phenotypes following SARS-CoV-2 infection. The neuropsychological profiles we identified in our study were highly heterogeneous, both cross-sectionally and over time. As suggested in the literature, we therefore suspected the existence of at least two clinical phenotypes. The first phenotype corresponds to the hypothesis that the long-term effects of SARS-CoV-2 infection are comparable to those of ME/CFS. As in ME/CFS, our results indicated the presence of executive and attentional deficits [11,12]. Moreover, our analyses aimed at identifying potential predictors of cumulative neuropsychological deficits indicated that changes in self-reported depressive symptoms were predictive of changes in executive functions. This could further argue in favor of a ME/CFS phenotype, as the literature describes a close association between neuropsychological deficits in this medical condition and psychiatric symptoms, especially anxiety and depression [11,12,49]. Some of our results were not congruent with what has been highlighted in ME/CFS to date. The executive and attentional deficits described above were accompanied by memory and instrumental disorders in our cohort. Moreover, with the exception of attentional deficits, the cumulative deficits persisted and even worsened, whereas the ME/CFS literature suggests an improvement in neuropsychological performance at six months [11]. Finally, GEE models failed to reveal any significant association between changes in self-reported fatigue and changes in cumulative neuropsychological deficits. That said, it is important to bear in mind that these statistical models are designed to study links between changes in status (in this case, changes in self-reported fatigue with changes in neuropsychological performances). If the levels remain, for example, stable and high in both conditions, the results are not significant. Moreover, our knowledge of the symptoms that accompany ME/CFS is still limited. We therefore believe that the hypothesis of an analogy between ME/CFS and post-COVID condition should be retained for the time being. Another, non-mutually exclusive explanation for the decline in performance in patients with no known pre-infection clinical history, as well as the presence of memory and instrumental deficits, would be the presence of a second phenotype where neurodegenerative processes are triggered by COVID-19 [6]. In particular, the increase in instrumental deficits (memory, language, and perception) observed in certain moderate and severe patients could be congruent at the behavioral level with an acceleration of neurodegenerative processes. Contradicting this hypothesis, few of the changes in cumulative neuropsychological deficits (memory, instrumental, attention) were predicted by psychiatric scores. One possible explanation lies in the use of self-reported questionnaires, which may underestimate the prevalence and the magnitude of the psychiatric symptoms in anosognosic patients. Further analyses according to phenotype, together with measures of biological markers, are needed to elucidate the heterogeneity of post-COVID-19 condition.

Our study had several main limitations. First, despite strict exclusion criteria, thorough screening of medical records and systematic interviews, a very small number of patients had comorbidities (respiratory or cardiovascular) that potentially influenced their long-term performance. Second, as with the previous study conducted at 6–9 months post-infection, we could not include a control group, owing to the high rate of infection in Geneva across 2020 and 2021. Given the scale of the pandemic, and unless a cohort was followed before the pandemic, no group in the world today could recruit such a control group, which is why we performed simulation analyses. Third, we performed said simulation analyses with just 95 patients, which may reduce the generalisability of the results. There may have been an overall tendency to overestimate the level of abnormality in each separate comparison [5]. That said, the simulation results were broadly comparable with what had previously been validated on 121 patients [6]. Moreover, we ran additional analyses with multivariate correction (i.e. Benjamini-Hochberg FDR). It is also important to acknowledge that the neuropsychological tests we used may have lacked sensitivity for some patients, possibly leading to an underestimation of their neuropsychological deficits. A sizeable percentage of patients in the mild group did not take part in the assessment at 12–15 months. The fact that so many patients with neuropsychological deficits at 6–9 months did not return may have influenced our results for cumulative deficits in the mild subgroup. Fourth, many potentially important variables, such as levels of vitamin D [50] and immunological markers [16,18] were not collected, even though they may play a role in the progression of neuropsychological symptoms following SARS-CoV-2 infection. Fifth, this study was carried out in a high-income country, so the question of cognitive long-term effects in low- and middle-income countries with socio-economic and cultural specificities remains unanswered [51].

## CONCLUSION

We identified several long-term neuropsychological trajectories 12–15 months after SARS-CoV-2 infection. These trajectories were partially independent of the severity of respiratory symptoms in the acute phase, and therefore cannot be attributed solely to post-ICU effects. Whereas the performances of some patients followed in the Geneva COVID-COG cohort had improved one year after being infected, a significant portion of patients displayed neuropsychological deficits that persisted, appeared or even increased over the long term. These trajectories could not be explained by changes in the psychiatric symptoms that may occur in the context of a global pandemic, such as PTSD or anxiety. Our study opens the way for the characterisation of specific phenotypes of the new neuropsychological syndrome that we have chosen to call neuropsychological post-COVID-19 condition.

## Additional material


Online Supplementary Document
