# Supplementary material for: Persistence and emergence of new neuropsychological deficits following SARS-CoV-2 infection: A follow-up assessment of the Geneva COVID-COG cohort
Source: J Glob Health. 2024 Mar 8;14:05008. doi: 10.7189/jogh.14.05008 (PMC10919907; doi:10.7189/jogh.14.05008)
Supplement: Online Supplementary Document [file jogh-14-05008-s001.pdf]

STROBE Statement—checklist of items that should be included in reports of observational studies

	Item No.	Recommendation	Page No.	Relevant text from manuscript
**Title and abstract**	1	(*a*) Indicate the study's design with a commonly used term in the title or the abstract	Page 1	
		(*b*) Provide in the abstract an informative and balanced summary of what was done and what was found	Page 3-4	
**Introduction**				
Background/rationale	2	Explain the scientific background and rationale for the investigation being reported	Pages 5-7	
Objectives	3	State specific objectives, including any prespecified hypotheses	Pages 7	
**Methods**				
Study design	4	Present key elements of study design early in the paper	Pages 8	
Setting	5	Describe the setting, locations, and relevant dates, including periods of recruitment, exposure, follow-up, and data collection	Page 8 and 14-15	
Participants	6	(*a*) *Cohort study*—Give the eligibility criteria, and the sources and methods of selection of participants. Describe methods of follow-up<br>*Case-control study*—Give the eligibility criteria, and the sources and methods of case ascertainment and control selection. Give the rationale for the choice of cases and controls<br>*Cross-sectional study*—Give the eligibility criteria, and the sources and methods of selection of participants	Cohort study: Page 8	
		(*b*) *Cohort study*—For matched studies, give matching criteria and number of exposed and unexposed<br>*Case-control study*—For matched studies, give matching criteria and the number of controls per case	Page 8	
Variables	7	Clearly define all outcomes, exposures, predictors, potential confounders, and effect modifiers. Give diagnostic criteria, if applicable	Pages 8-13	
Data sources/ measurement	8*	For each variable of interest, give sources of data and details of methods of assessment (measurement). Describe comparability of assessment methods if there is more than one group	Pages 8-13	
Bias	9	Describe any efforts to address potential sources of bias	Page 8	
Study size	10	Explain how the study size was arrived at	Page 14-15	

Continued on next page

Quantitative variables	11	Explain how quantitative variables were handled in the analyses. If applicable, describe which groupings were chosen and why	Page 11-13
Statistical methods	12	(*a*) Describe all statistical methods, including those used to control for confounding	Page 11-13
		(*b*) Describe any methods used to examine subgroups and interactions	Page 11-13
		(*c*) Explain how missing data were addressed	Patients with missing data excluded (page 14)
		(*d*) *Cohort study*—If applicable, explain how loss to follow-up was addressed<br>*Case-control study*—If applicable, explain how matching of cases and controls was addressed<br>*Cross-sectional study*—If applicable, describe analytical methods taking account of sampling strategy	Page 14
		(*e*) Describe any sensitivity analyses	Not applicable
**Results**			
Participants	13*	(a) Report numbers of individuals at each stage of study—eg numbers potentially eligible, examined for eligibility, confirmed eligible, included in the study, completing follow-up, and analysed	Page 14
		(b) Give reasons for non-participation at each stage	Page 14
		(c) Consider use of a flow diagram	Page17
Descriptive data	14*	(a) Give characteristics of study participants (eg demographic, clinical, social) and information on exposures and potential confounders	Page 14-15
		(b) Indicate number of participants with missing data for each variable of interest	No missing data
		(c) *Cohort study*—Summarise follow-up time (eg, average and total amount)	Page 14
Outcome data	15*	*Cohort study*—Report numbers of outcome events or summary measures over time	Page 14
		Case-control study—Report numbers in each exposure category, or summary measures of exposure	-
		Cross-sectional study—Report numbers of outcome events or summary measures	-
Main results	16	(*a*) Give unadjusted estimates and, if applicable, confounder-adjusted estimates and their precision (eg, 95% confidence interval). Make clear which confounders were adjusted for and why they were included	-
		(*b*) Report category boundaries when continuous variables were categorized	Page 8-9
		(*c*) If relevant, consider translating estimates of relative risk into absolute risk for a meaningful time period	-

Continued on next page

Other analyses	17	Report other analyses done—eg analyses of subgroups and interactions, and sensitivity analyses	Page 11-14
**Discussion**			
Key results	18	Summarise key results with reference to study objectives	Page 26
Limitations	19	Discuss limitations of the study, taking into account sources of potential bias or imprecision. Discuss both direction and magnitude of any potential bias	Page 28-29
Interpretation	20	Give a cautious overall interpretation of results considering objectives, limitations, multiplicity of analyses, results from similar studies, and other relevant evidence	Pages 26-28
Generalisability	21	Discuss the generalisability (external validity) of the study results	Page 28-29
**Other information**			
Funding	22	Give the source of funding and the role of the funders for the present study and, if applicable, for the original study on which the present article is based	Page 31

*Give information separately for cases and controls in case-control studies and, if applicable, for exposed and unexposed groups in cohort and cross-sectional studies.

Note: An Explanation and Elaboration article discusses each checklist item and gives methodological background and published examples of transparent reporting. The STROBE checklist is best used in conjunction with this article (freely available on the Web sites of PLoS Medicine at http://www.plosmedicine.org/, Annals of Internal Medicine at http://www.annals.org/, and Epidemiology at http://www.epidem.com/). Information on the STROBE Initiative is available at www.strobe-statement.org.

Table S1. Percentages of self-reported symptoms in acute phase according to severity of respiratory symptoms

	Total Sample (%) *N* = 95	Mild subgroup (%) *n* = 33	Moderate subgroup (%) *n* = 39	Severe subgroup (%) *n* = 23
Runny nose	25.26	36.36	17.95	21.74
Sore throat	14.74	21.21	12.82	8.70
Muscle pain	48.42	72.73	33.33	39.13
Loss of sense of smell	45.26	63.64	48.72	13.04
Taste disorder	45.26	63.64	48.72	13.04
Dry cough	53.68	51.52	56.41	52.17
Productive cough	6.32	15.15	2.56	0.00
Fever	67.37	57.58	74.36	69.57
Digestive symptoms	34.74	39.39	38.46	21.74
Fatigue	78.95	87.88	82.05	60.87
Difficulty breathing	45.26	33.33	53.85	47.83
Chest pain	21.05	21.21	20.51	21.74
Headache	58.95	81.82	53.85	34.78
Somnolence	26.32	58.95	28.21	13.04
Nonrestorative sleep	35.79	39.39	38.46	26.09
Insomnia	16.84	12.12	17.95	21.74
Waking up feeling choked or suffocated	13.68	9.09	17.95	13.04
Snoring	1.05	0.00	2.56	0.00
Interruption of breathing during sleep	4.21	0.00	5.13	8.70
Other	18.95	18.18	25.64	8.70
None	4.21	3.03	0.00	13.04

Note. Mild: patients not hospitalized for SARS-CoV-2 infection; Moderate: patients hospitalized without mechanical ventilation for SARS-CoV-2 infection; Severe: patients hospitalized in intensive care with mechanical ventilation for SARS-CoV-2 infection.

Table S2. Cumulative percentages of abnormally low neuropsychological scores for whole sample and each subgroup (mild, moderate, and severe) at 6–9 and 12-15 months post-infection and those estimated for a normative population

			Frequency (%) of impaired scores at 6-9 months				Frequency (%) of impaired scores at 12-15 months			
		**Estimated cumulative percentages for normative population at 6-9 months (< 5th percentile)**	**Whole sample *N* = 95**	**Mild *n* = 33**	**Moderate *n* = 39**	**Severe *n* = 23**	**Whole sample *N* = 95**	**Mild *n* = 33**	**Moderate *n* = 39**	**Severe *n* = 23**
Perception (4 tests)	0 low scores	82.19%	84.21%	84.85%	84.62%	82.61%	86.32%	93.94%	87.18%	73.91%
	≥ 1 low score	17.81%	15.79%	15.15%	15.38%	17.39%	13.68%	6.06%	12.82%	26.08%[@]
	≥ 2 low scores	1.98%	3.16%	0%	5.13%[@]	4.35%[@]	1.05%	0%	0%	4.35%[@]
	≥ 3 low scores	0.14%	0%		0%	0%	1.05%			4.35%[@]
	4 low scores	0.00%					0%			0%
Ideomotor praxis (3 tests)	0 low scores	86.61%	90.53%	87.88%	94.87%	86.96%	97.89%	96.97%	97.44%	100.00%
	≥ 1 low scores	13.39%	9.47%	12.12%	5.13%	13.04%	2.11%	3.03%	2.56%	0%
	≥ 2 low scores	1.55%	0%	0%	0%	0%	0%	0%	0%	
	3 low scores	0.10%								
Language (5 tests)	0 low scores	80.27%	86.32%	87.88%	89.74%	78.26%	85.26%	87.87%	84.62%	82.61%
	≥ 1 low score	19.73%	13.68%	12.12%	10.26%	21.74%	14.74%	12.12%	15.38%	17.39%
	≥ 2 low scores	4.30%	0%	0%	0%	0%	1.05%	0%	2.56%	0%
	≥ 3 low scores	0.87%					1.05%		2.56%[@]	
	≥ 4 low scores	0.14%					0%		0%	
	5 low scores	0.01%								
Executive functions (11 tests)	0 low scores	67.57%	72.63%	81.82%	66.67%	69.57%	78.95%	87.88%	79.49%	65.22%
	≥ 1 low score	32.43%	27.37%	18.18%	33.33%	30.43%	21.05%	12.12%	20.51%	34.78%[@]
	≥ 2 low scores	12.62%	9.47%	6.06%	12.82%	8.70%	9.47%	0%	15.38%[@]	13.04%
	≥ 3 low scores	5.61%	4.21%	0%	7.69%[@]	4.35%	3.16%		5.13%	4.35%
	≥ 4 low scores	2.64%	0%		0%	0%	1.05%		0%	4.35%[@]
	≥ 5 low scores	1.17%					1.05%			4.35%[@]
	≥ 6 low scores	0.47%					0%			0%
	≥ 7 low scores	0.16%								
	≥ 8 low scores	0.04%								
	≥ 9 low scores	0.01%								
	≥ 10 low scores	0.00%								
	11 low scores	0.00%								
Attentional functions	0 low scores	64.60%	73.68%	81.82%	66.67%	73.91%	80.00%	84.84%	74.36%	82.61%
	≥ 1 low score	35.40%	26.32%	12.12%	30.08%	22.79%	20.00%	15.15%	25.64%	17.39%

(10 tests)	≥ 2 low scores<br>≥ 3 low scores<br>≥ 4 low scores<br>≥ 5 low scores<br>≥ 6 low scores<br>≥ 7 low scores<br>≥ 8 low scores<br>≥ 9 low scores<br>10 low scores	10.92%<br>2.85%<br>0.60%<br>0.10%<br>0.02%<br>0.00%<br>0.00%<br>0.00%<br>0.00%	13.68%<br>6.32%<br>4.21%<br>1.05%<br>0%	6.06%<br>3.03%<br>0%	9.57%<br>1.88%<br>1.88%@<br>0.83%<br>0%	14.10%@<br>1.05%<br>0%	6.31%<br>1.05%<br>0%	0%	7.69%<br>0%	13.04%<br>4.35%@<br>0%
Memory<br>(8 tests)	0 low scores<br>≥ 1 low score<br>≥ 2 low scores<br>≥ 3 low scores<br>≥ 4 low scores<br>≥ 5 low scores<br>≥ 6 low scores<br>≥ 7 low scores<br>8 low scores	75.64%<br>24.36%<br>9.82%<br>3.73%<br>1.45%<br>0.52%<br>0.15%<br>0.04%<br>0.01%	69.47%<br>30.53%@<br>10.53%<br>4.21%<br>1.05%<br>1.05%@<br>0%	88.24%<br>11.76%<br>5.88%<br>2.94%<br>0%	60.53%<br>39.47%@<br>10.53%<br>0%	56.52%<br>43.48%@<br>17.39%@<br>13.04 %@<br>4.35%@<br>4.35%@<br>0%	76.84%<br>23.16%<br>7.37%<br>4.21%<br>2.11%<br>0%	90.91%<br>9.09%<br>0%	87.18%<br>12.82%<br>7.69%<br>5.12%@<br>0%	60.87%<br>39.13%@<br>13.04%@<br>4.35%@<br>4.35%@<br>0%
Logical reasoning<br>(2 tests)	0 low scores<br>≥ 1 low score<br>2 low scores	91.50%<br>8.50%<br>1.51%	98.95%<br>1.05%<br>1.05%	100%<br>0%	97.44%<br>2.56%<br>2.56%@	100%<br>0%	98.95<br>1.05%<br>1.05%	100%<br>0%	97.34%<br>2.56%<br>2.56%@	100%<br>0%

Note. @ significant after FDR correction compared with estimate for normative population.

Commented [IHJDA1]: Faudrait que ce soit un * normalement selon les guidelines
